# Transcranial Alternating Current Stimulation of the Primary Motor Cortex after Skill Acquisition Improves Motor Memory Retention in Humans: A Double-Blinded Sham-Controlled Study

**DOI:** 10.1093/texcom/tgaa047

**Published:** 2020-08-06

**Authors:** Tomofumi Yamaguchi, Christian Svane, Christian Riis Forman, Mikkel Malling Beck, Svend Sparre Geertsen, Jesper Lundbye-Jensen, Jens Bo Nielsen

**Affiliations:** 1 Department of Neuroscience, University of Copenhagen, 2200 Copenhagen, Denmark; 2 Department of Physical Therapy, Faculty of Health Science, Juntendo University, Tokyo 113-8421, Japan; 3 Department of Nutrition, Exercise and Sports, University of Copenhagen, 2200 Copenhagen, Denmark; 4 Elsass Foundation, 2920 Charlottenlund, Denmark

**Keywords:** beta-band stimulation, corticomuscular coherence, intramuscular coherence, motor memory consolidation, transcranial alternating current stimulation

## Abstract

Consolidation leading to retention of motor memory following motor practice involves activity-dependent plastic processes in the corticospinal system. To investigate whether beta-band transcranial alternating current stimulation (tACS) applied immediately following skill acquisition can enhance ongoing consolidation processes and thereby motor skill retention 20 adults participated in a randomized, double-blinded, sham-controlled study. Participants received tACS at peak beta-band corticomuscular coherence (CMC) frequency or sham tACS for 10 min following practice of a visuomotor ankle dorsiflexion task. Performance was measured as the average percentage time on target. Electroencephalograhy (EMG) was measured at Cz and EMG from the right tibialis anterior muscle. CMC and intramuscular coherence (IMC) were estimated during 2-min tonic dorsiflexion. Motor skill retention was tested 1 and 7 days after motor practice. From the end of motor practice to the retention tests, motor performance improved more in the tACS group compared with the sham tACS group after 1 (*P* = 0.05) and 7 days (*P* < 0.001). At both retention tests, beta-band IMC increased in the tACS group compared with post-tACS. Beta-band CMC increased in the tACS group at retention day 1 compared with post-tACS. Changes in CMC but not IMC were correlated with performance 1 and 7 days following practice. This study shows that tACS applied at beta-band CMC frequency improves consolidation following visuomotor practice and increases beta-band CMC and IMC. We propose that oscillatory beta activity in the corticospinal system may facilitate consolidation of the motor skill.

## Introduction

Throughout life, skilled motor control is vital for interaction with the environment and is heavily dependent on descending cortical control of the muscles. When the descending cortical control improves through motor skill learning, it is accompanied by both increases in the excitability (Perez et al. 2004) and in the functional oscillatory coupling of the corticospinal drive to motor neurons (Perez et al. 2006). Transcranial alternating current stimulation (tACS) is a noninvasive brain stimulation technique that employs oscillatory electrical stimulation alternating between 2 electrodes at a predetermined frequency. tACS is speculated to alter the power of oscillatory brain rhythms through synchronization of neuronal networks (Ali et al. 2013) and is able to increase corticospinal excitability especially through beta-range stimulations with intensity above 1 mA (Wischnewski et al. 2019). Some studies have indicated a potentiating effect of beta-range tACS on motor sequence performance memory through an effect on early consolidation (Krause et al. 2016) yet the relation to neurophysiological measures still remains to be explored.

The functional oscillatory coupling between the cortical motor areas and the spinal motor neurons of active muscles is reflected in both corticomuscular and intramuscular coherence (CMC and IMC, respectively) (Grosse et al. 2002). CMC and IMC are both measures of the association between the descending cortical drive and the muscle activity. However, whereas CMC can be considered a direct assessment of the association between cortical activity and muscle activity, IMC indirectly infers common synaptic input to the motor neuron pool of the muscle which can be of cortical origin, but can also include other common synaptic inputs to the spinal motor neurons. The measures therefore complement each other but can also add further information about the neural activity underlying motor behavior (Grosse et al. 2002).

When acquiring new motor skills, changes in coherence measures have been observed in both healthy individuals (Perez et al. 2006; Masakado and Nielsen 2008) and individuals with central motor lesions (Belardinelli et al. 2017). Coherence is evident at beta-band frequencies during static contractions and at gamma band frequencies during dynamic movement (Conway et al. 1995; Omlor et al. 2007; Spedden et al. 2019). Both these frequency bands are related to activity originating in the primary motor cortex (Conway et al. 1995; Grosse et al. 2002; Hansen and Nielsen 2004). Increased CMC and IMC at beta-band frequencies have been found in the trained, but not in the untrained muscle following motor practice (Perez et al. 2006), and changes in beta-band power in the primary motor cortex (M1) have also been observed during early motor memory consolidation (Espenhahn et al. 2019).

Therefore, since beta-band stimulations can potentially both increase the corticospinal excitability and modulate the oscillatory power of ongoing motor cortical activity, we hypothesized that beta-band tACS can facilitate early consolidation processes and thereby enhance retention of motor memory. We further investigated whether tACS results in an increased functional oscillatory coupling between M1 and the spinal motor neurons in the applied frequency band and whether this may be related to effects on retention of motor memory.

## Materials and Methods

### Participants

Twenty able-bodied individuals participated in this study (10 female, aged 24.4 ± 2.1 [mean ± standard deviation {SD}] years). All participants gave written informed consent and received monetary compensation for their participation. The study was approved by the ethics committee of the Greater Copenhagen area and performed in accordance with the Declaration of Helsinki. No participants reported a history of neurological and/or orthopedic diseases or were being treated with medication affecting the central nervous system. All participants were right leg dominant according to the foot preference test (Chapman and Chapman 1987). The number of participants was estimated based on a previous study (effect size dz = 1.15) demonstrating a relationship between motor skill learning and changes in EEG–EMG coherence (Perez et al. 2006).

### Experimental Procedure

We used a randomized, double-blinded, sham-controlled study design. Participants were stratified by sex and randomly allocated to receive tACS (real tACS) or sham tACS (sham tACS). After motor practice, a computer-generated list randomly assigned the allocation into the 2 groups. To ensure double blinding, the experimenter in charge of the experiment and the data analysis (T.Y.) was not in possession of the list. Instead, 2 other experimenters (C.F. or C.S.) controlled the application of real or sham stimulations.

**Figure 1:**
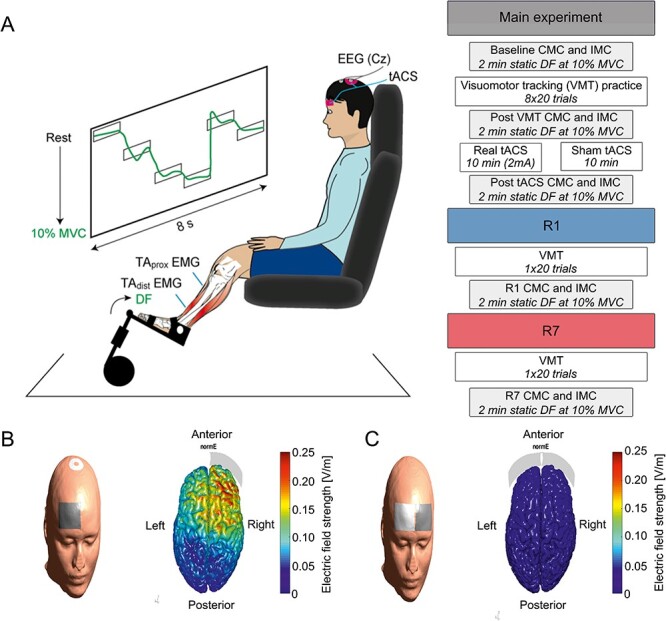
Experimental setup and procedure. (*A*) Participants performed the motor task using varying force levels of dorsiflexion up to 10% of MVC. The time on target score is calculated as the percentage of time that the green line is within the target boxes. During motor practice, each trial lasted 8 s and 20 trials were performed per block. EMG was measured distally and proximally on the anterior tibial (TA) muscle and EEG was measured from the vertex (Cz) at 5 time points: baseline, post-VMT practice, post-tACS/sham intervention, at R1, and at R7. Simulated, putative norm electric fields for the real (*B*) and sham (*C*) tACS using SimNIBS2.1 with example subject “Ernie” as template (www.simnibs.org; http://dx.doi.org/10.1101/500314). DF = dorsiflexion; VMT = visuo-motor tracking.

All participants performed a 42 min session of motor practice consisting of 8 blocks of each 20 trials involving continuous dorsiflexion of the right ankle (Fig. 1*A*). After motor practice, participants received 10 min of tACS or sham tACS. Retention tests 1 day (R1) and 7 days (R7) after the intervention consisted of a single block of 20 trials. Electroencephalograhy (EEG) and electromyography (EMG) were recorded during bouts of 2 min isometric contractions of the ankle dorsiflexors at 10% maximum voluntary contraction (MVC) before and after motor practice, immediately after tACS or sham tACS, 1 day after (R1) and 7 days after (R7) motor practice. EMG was recorded from both the proximal and distal part of the Tibialis anterior (TA) muscle to enable IMC analyses. CMC analyses were performed using EEG and EMG from the proximal TA.

**Table 1:** Participant characteristics

	Real tACS	Sham tACS
No. of participants	10	10
Sex (F/M)	5/5	5/5
Age (years)	24.1 ± 1.7	24.7 ± 2.5
Footedness (R/L)	10/0	10/0
MVC (Nm)	205.3 ± 80.0	228.7 ± 77.4

Note: Table 1 presents participant characteristics. No significant differences were observed in any measure between groups. Data are presented as means ± SD.

### Transcranial Alternating Current Stimulation

tACS was administered using a DC STIMULATOR PLUS (NeuroConn, Ilmenau, Germany). Three electrodes were placed similarly during real and sham tACS stimulations. A ring electrode (inner radius = 0.75 cm, outer radius = 2.25 cm, area = 14.13 cm^2^) was placed over the leg area of the motor cortex contralateral to the investigated muscle. Two frontal electrodes (7 × 5 cm) were placed at the left and right orbital (Fig. 1). For the real stimulation, tACS was delivered at 2 mA (peak-to-peak-amplitude; sinusoidal waveform) between the ring electrode and right frontal electrode for 10 min while the participant was at rest. Electrical field simulations (SimNIBS2: www.simnibs.org; http://dx.doi.org/10.1101/50031) of a current strength of 1 mA (corresponding to the 2 mA peak-to-peak amplitude) showed that this electrode arrangement induced an electrical field covering a bilateral area around Cz including the representation of the right leg muscles in the left primary motor cortex using the exemplary “Ernie” head model provided in the SimNIBS simulation tool (Fig. 1*B*). The stimulation frequency was individually targeted at the EEG–EMG peak beta-band frequency following motor practice, as increases in this frequency band have been associated with increases in visuomotor task performance (Perez et al. 2006). It was hypothesized that the depolarizations of the tACS stimulation in this way would have the best conditions for potentiating the ongoing consolidation processes. For the sham stimulation, the same procedure was used, but the stimulation was delivered at 0.5 mA between the frontal electrodes (Fig. 1*C*). This was done to replicate phosphenes: a perception of visual flicker that is due to retinal stimulation. Phosphenes are especially common when tACS is used at beta and gamma frequencies (Turi et al. 2013). At the end of the first day (i.e., the main experiment), participants filled out a questionnaire in which they answered whether they thought they had received real stimulation, sham stimulation, or did not know.

### Motor Practice

All participants performed a 42-min session of motor practice involving visuomotor accuracy tracking with rapid shifts in force levels. The task was identical to the task employed by Christiansen et al. (2018), but involved precise control of ankle dorsiflexion force. The training consisted of 8 blocks of 3.3 min isometric ankle dorsiflexion activity with 2 min of rest in between. Each block consisted of 20 individual trials. During each trial, participants controlled the vertical position of a cursor on the screen by performing continuous ankle dorsiflexion movements (Fig. 1*A*), while the cursor moved horizontally across the monitor (left to right) in 8 s. Dorsiflexion made the cursor move downward. After each trial, the participant received a score between 0 and 100 representing the relative amount of time in which the cursor was inside the desired boxes (i.e., time on target). The motor practice was performed using the dominant, right leg.

### E‌EG and EMG Recordings

EEG recordings were obtained using a bipolar silver electrode-pair montage. The electrodes were placed at the vertex (Cz) and 5 cm frontal to Cz (Perez et al. 2006) as studies have established that CMC is focalized around Cz for the ankle muscles (Petersen et al. 2012; Spedden et al. 2018). EMG was obtained from two pairs of surface electrode placed proximally and distally on the tibialis anterior muscle (TA) of the dominant, right leg. EEG signals were amplified × 10 000 and EMG signals × 1000, and both signals were band-pass filtered (1–1000 Hz) using a Neurolog amplifier system (Digitimer, Hertfordshire, UK). All electrode positions were marked and electrodes were carefully repositioned for recordings on different days. A similar low impedance was ensured for electrodes during the recordings. Both EEG and EMG signals were sampled at 5000 Hz using a 1401 AD board and Spike 2.0 software (Cambridge Electronics Design Ltd, Cambridge, UK).

### Data Analysis and Statistics

All the electrophysiological data analyses were conducted in MATLAB^®^ (Version 2016b, MathWorks, MA, USA). Calculation of the frequency-domain correlation between EEG and EMG signals was estimated using the coherence function |*R_xy_*(*λ*)|. Coherence was calculated between the EEG signal and the TA EMG signal from the proximal electrodes, probing CMC, and as IMC from comparison of the proximal and distal TA EMG-signals. The approximation procedure has been thoroughly described elsewhere (Halliday et al. 1995). In brief, auto-spectra and cross-spectra were computed by isolating signals into nonoverlapping data segments. Subsequently, Fourier transformations were performed on these segments and averaged. The coherence spectrum was then computed from the squared cross-spectrum normalized to the product of the 2 autospectra:

(1) \begin{equation*} {\left|{R}_{xy}\left(\lambda \right)\right|}^2=\frac{{\left|{f}_{xy}\left(\lambda \right)\right|}^2}{f_{xx}\left(\lambda \right)\ {f}_{yy}\left(\lambda \right)} \end{equation*}

Serving as a frequency-specific parallel to the correlation coefficient, the coherence measure represents an index of the linear association between electrophysiological signals from cortex and muscle quantified as a value between 0 and 1. Each recording lasted 120 s in duration and the sampling frequency was 5000 Hz. The segment length was 4096, giving 146 segments per recording with a spectral resolution of 1.22 Hz.

To perform groupwise comparisons of coherence, the individual coherence estimates were combined into a single summary coherence estimate, the pooled coherence, which can be interpreted in the same way as an individual coherence measure (Amjad et al. 1997). This was performed by pooling the individual second order spectra. The statistical difference of coherence between groups was performed by the chi-squared difference of coherence test, which tests the null hypothesis of equal coherence (Amjad et al. 1997). To evaluate the significance of coherence, an upper 95% confidence interval (CI) was computed.

The remaining statistical analyses were carried out using R Studio (R Core Team, Vienna, Austria). Potential between-group baseline differences in participant characteristics (e.g., age and gender) were compared using unpaired *t*-tests and chi-squared tests for continuous and categorical data, respectively. To determine the effects of tACS on motor performance, we investigated potential group differences over time in the visuo-motor tracking (VMT) task by means of fitted two-way linear mixed models using the R package *lme4* (Bates et al. 2014). Intervention group and time were added with an interaction term to a global model as fixed effects (group × time). To account for potential effects related to interindividual differences, we included the individual participants as random intercepts. Model validation was confirmed by visual inspection of probability plots and residual plots to evaluate normality and homogeneity of residuals, respectively. To accommodate the specific hypotheses of this study, specific sets of contrasts between intervention groups across, and at, the included time-points were computed. Delta values for total learning were calculated as absolute differences between time points (i.e., R1-Block 1 and R7-block 1).

Subsequently, model-based *t*-tests were used to identify the specific differences, using the R-package *multcomp* (Hothorn et al. 2008). The pairwise comparisons were adjusted using the “single-step” method. Model-based estimates are reported as mean $\pm$ standard error of the estimate, unless otherwise stated. Familywise 95% confidence intervals are reported when appropriate. Analyses of potential correlations between acquisition and retention of motor memory and between retention and changes in coherence measures were performed as Pearson product moment correlation tests. Outlier values were defined as being more than 2.5 standard deviations from the group mean and removed prior to the analyses. Data in figures are reported as mean values. For all analyses, a significance level of 0.05 was applied.

## Results

### Participant Characteristics and Baseline Motor Performance

**Figure 2:**
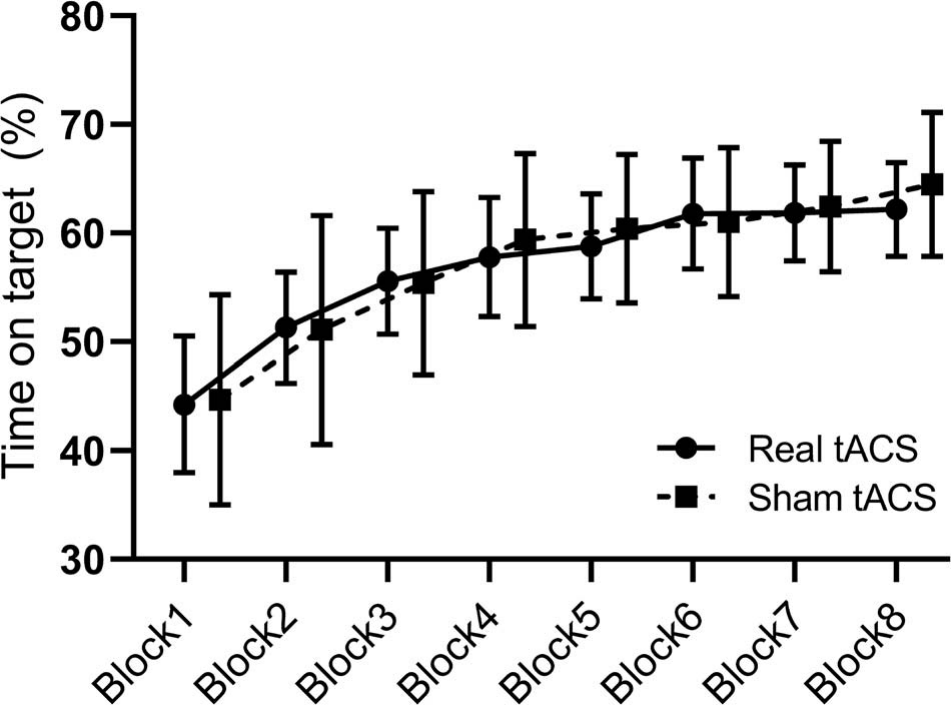
Motor skill acquisition. This figure shows the group average time on target scores for the real tACS group (black circles, solid line) and the sham tACS group (black squares, dashed line) in all 8 motor practice blocks. Each group average is presented with 95% confidence intervals.

The groups were not significantly different in terms of participants’ age, dorsiflexion MVC, footedness or sex distributions (all *P*-values > 0.05). Questionnaire of the blinding for tACS stimulation showed that participants correctly identified real stimulation in 20% of cases, while sham stimulation was correctly recognized in 30% of cases. As the correct answer of stimulation condition was below chance level, the blinding procedure was considered successful. Performance of the VMT task was evaluated as time on target, representing the percentage of time in which the cursor was inside the targets. The time on target was not found to significantly differ at baseline prior to training in the 2 groups (*P* = 0.92) (Fig. 2). One participant in the sham tACS group experienced a large drop in performance from block8 to R1 (>2.5 SD away from group mean). Therefore, data from this subject were removed from the R1 analysis.

### No Significant Group Differences in Performance Following Practice

To determine whether the participants acquired the task, and whether they did so to the same extent, we evaluated within and between-group differences in the online gains, that is, by comparing practice block 8 with practice block 1. First, we found that both groups improved performance from practice block 1 to block 8 (real tACS: 17.9 ± 1.9%, *P* < 0.001; sham tACS: 19.8 ± 1.9%, *P* < 0.001). Importantly, no significant difference was found in the acquisition of the motor task following visuomotor training between the 2 groups: 1.8 ± 2.8% (*P* = 0.50, CI [−7%, 3.5%]) (Fig. 2).

### Beta-Band tACS Improves Visuomotor Skill Retention

**Figure 3:**
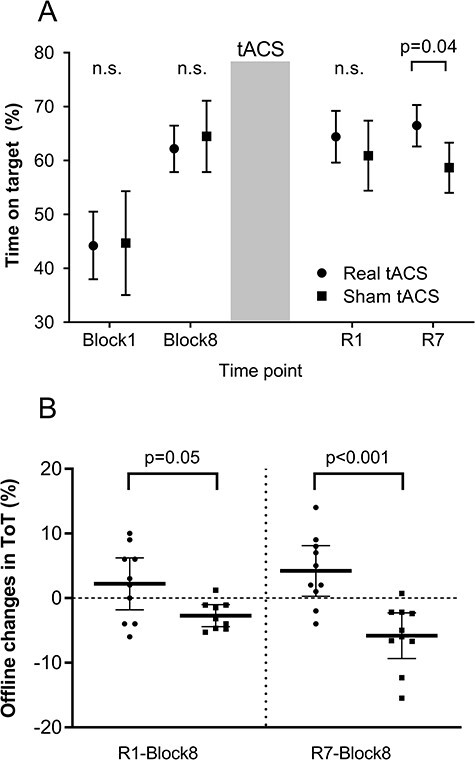
Motor skill retention. (*A*) Average time on target score for the real tACS group (black dots) and the sham tACS group (black squares) in the first motor practice block, the last motor practice block (block 8), the day 1 retention test (R1), and the day 7 retention test (R7). The gray area dividing block 8 and R1 represents the intervention stimulation. (*B*) Change in time on target score for each group from block 8 to R1 and from block 8 to R7. The overlaid black lines show the group average (thick line) with the 95% confidence intervals (thin error lines). One participant was removed from the sham tACS group in this analysis, as the participant underperformed on the R1 test compared with the group average (offline change from block 8 to R1 was >2.5 SDs lower than the group’s average).

Performance in the motor task increased overnight in the real tACS group, whereas it decreased in the sham tACS group (Fig. 3*A*,*B*). In fact, the changes in motor performance from block 8 to R1 were marginally greater in the real tACS group compared with the sham tACS group 5.0% $\pm$ 2.6% (*P* = 0.05, 95% CI [−0.1% 10.1%]) and significantly greater from Block 8 to R7 10.0% $\pm$ 2.6% (*P* < 0.001, 95% CI [4.9% 15.1%]) [Fig. 3*B*]. These different offline changes in performance resulted in the tACS group outperforming the sham tACS group at R7 by 8.6% $\pm$ 4.2% (*P* = 0.04, CI [0.42%, 16.7%]), but not at R1 3.5% $\pm$ 4.2% (*P* = 0.4) (Fig. 3*A*). Thus, beta-band tACS applied after visuomotor training improved visuomotor skill retention.

**Figure 4:**
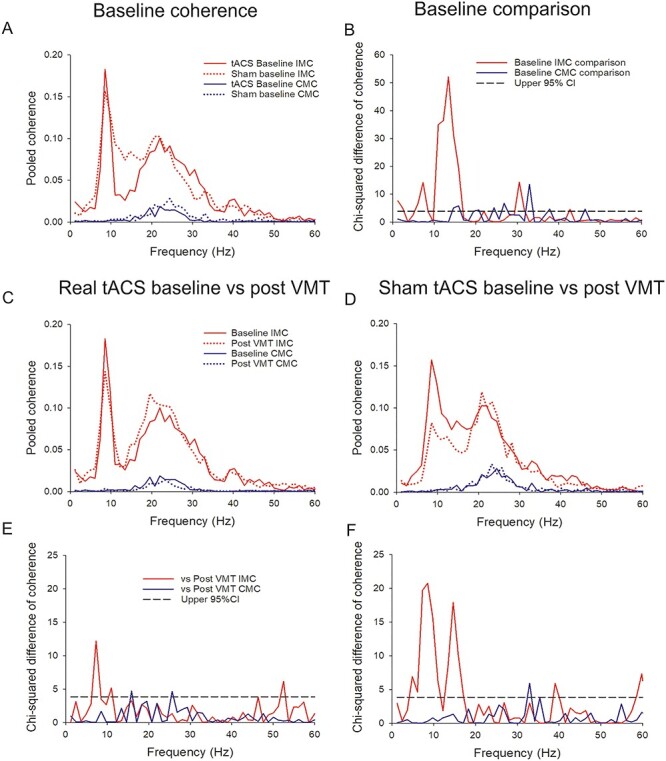
CMC and IMC at baseline and post-VMT. (*A*) presents baseline pooled coherence values for the real tACS group (solid lines) and the sham tACS group (dotted lines) for both IMC (red) and CMC (blue). (*B*) presents the comparison of pooled coherence between real tACS group and sham tACS group at baseline for IMC (red) and CMC (blue). In (*C*) and (*D*), red lines represent IMC, blue lines CMC, solid lines coherence at baseline and dotted lines after the 8 blocks of VMT practice in the real (*C*) and Sham (*D*) tACS groups. (*E* and *F*) Corresponding chi-squared difference of coherence tests for IMC (red) and CMC (blue).

**Figure 5:**
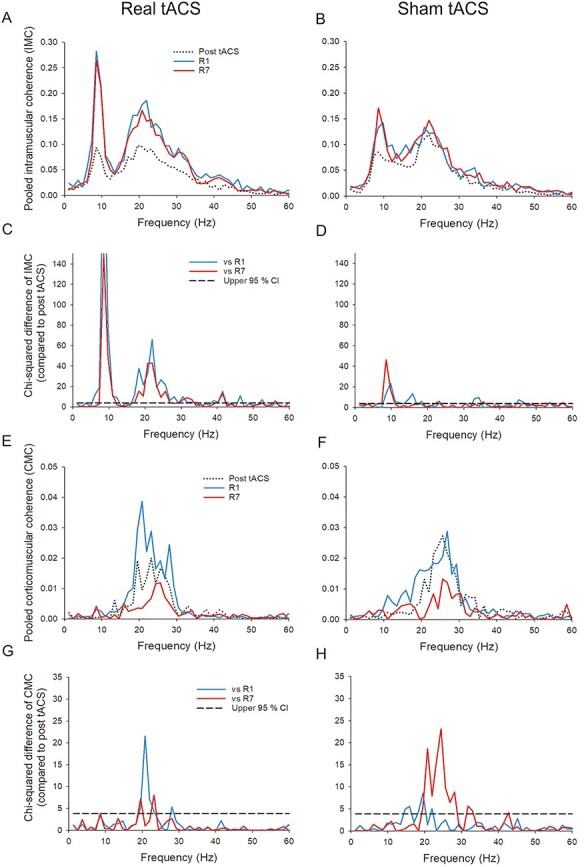
Coherence after real or sham tACS and at retention day 1 (R1) and 7 (R7) compared with post-tACS. Intramuscular pooled coherence (*A* and *B*) and corticomuscular pooled coherence (*E* and *F*) and intramuscular chi-squared difference of coherence test (*C* and *D*) and corticomuscular chi-squared difference of coherence test (*G* and *H*). The coherence values of the group which received real tACS are presented on the figures left side (*A* and *E*) and the group which received sham tACS are presented on the figures right side (*B* and *F*) from 3 time points; immediately after the applied tACS intervention (dotted black line), the R1 (solid blue line), and the R7 (solid red line). The 4 chi-squared differences of coherence tests (*C*, *D*, *G*, *H*) show individual difference of coherence comparisons between the post-tACS time point and R1 (solid blue line), and R7 (solid red line). In (*C*, *D*, *G*, and *H*), the black dashed line illustrates the upper 95% confidence limit under the null hypothesis of equal coherences.

### CMC and IMC

Figure 4*A* and *B* shows the pooled coherence measures at baseline for both groups (Fig. 4*A*) and a comparison of IMC and CMC baseline values between the real tACS group and the sham tACS group (Fig. 4*B*). All subjects had significant beta IMC at baseline, and 9 out of 10 in each group had significant CMC. A difference at baseline was observed for the IMC in the late alpha and early beta band (10–17 Hz) where the sham tACS IMC was higher. Figure 4*C*–*F* presents the real tACS group (Fig. 4*C*) and Sham tACS group (Fig. 4*D*) at baseline and following the 8 blocks of motor practice. The chi-squared difference of coherence tests (Fig. 4*E* and *F*) showed that the main difference in the real tACS group was a significant decrease in IMC low alpha-band frequency (7 Hz) from baseline to post visuomotor training (Fig. 4*C*). The main differences in the sham tACS group were also found to be significant decreases in the IMC, particularly the alpha and low beta-band frequencies (5–11 Hz and 13–17 Hz).

Figure 5*A*, *B* and *E*, *F* presents the pooled coherence analyses at 3 different time points: After the tACS intervention (“post tACS”) and at the 2 retention tests: R1 and R7. Figure 5*C*, *D* and *G*, *H* further presents the difference of coherence tests between the post-tACS, and the 2 retention tests, respectively. The IMC of the tACS group was increased in the 8–10 Hz and the 16–26 Hz frequency band at R1 and R7 compared with post-tACS (Fig. 5*A* and *C*). The IMC of the sham tACS group (Fig. 5*B* and *D*) was mainly found to significantly increase in the 9–11 Hz frequency band but also showed an increase in the 14–16 Hz frequency band at the R1 test compared with post-tACS. The CMC of the tACS group was significantly increased in the 20–23 Hz frequency band at R1, whereas the main change in CMC of the sham tACS group was a significant decrease in the 19–29 Hz frequency band compared with post-tACS.

### Correlation Tests

**Figure 6:**
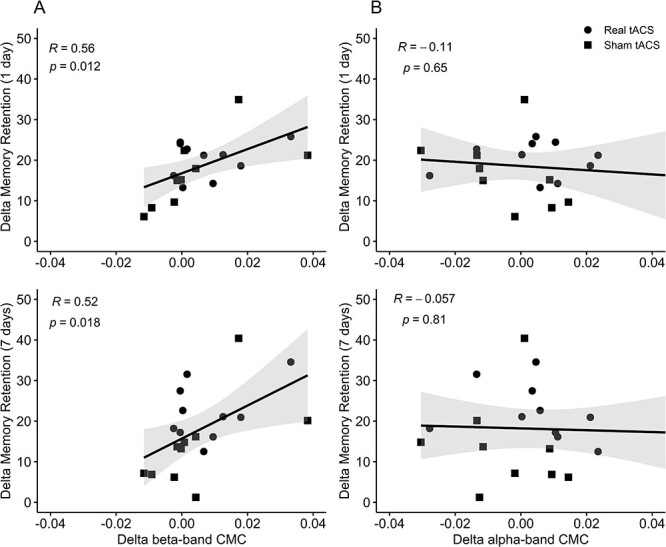
Correlations between changes in corticomuscular coherence and skill learning. Scatter plot and regression line representing Pearson Product correlations between changes from after tACS to R1 in corticomuscular coherence in the beta (*A*) and alpha band (*B*) and total retention at 1 day (top) 7 days (bottom).

In order to investigate potential relations between retention of motor memory and the observed changes in coherence parameters, restricted correlation tests were performed between these measures across all participants. Due to the considerable number of possible associations between parameters, and the aim of the present paper, the correlation analyses were restricted to investigate only (1) whether within-session changes in motor performance from baseline to after practice were related performance at R1 (*r* = 0.76, *P* < 0.001) and R7 (*r* = 0.68, *P* = 0.002) and (2) whether changes in alpha and beta-band CMC and IMC between sessions were related to delayed retention of motor performance. Offline changes in CMC from the intervention to R1 positively correlated with total retention of motor memory 1 day (*r* = 0.56, *P* = 0.012) and 7 days later (*r* = 0.52, *P* = 0.018) (Fig. 6). On the other hand, no significant correlations were found between changes in alpha-band CMC and total retention of motor memory at R1 (*r* = −0.11, *P* = 0.65) or R7 (*r* = −0.06, *P* = 0.81) (Fig. 6). No significant correlations with performance improvements were observed for the offline changes in IMC.

## Discussion

The aim of this study was to investigate the effects of tACS on the consolidation processes following visuomotor skill acquisition. We hypothesized that the alternating depolarizations of tACS in the beta-band frequency over the primary motor cortex would potentiate ongoing motor memory consolidation processes in the corticospinal motor networks following motor practice. We found that beta-band frequency tACS applied following visuomotor skill training enhanced retention of the acquired visuomotor skill compared with sham tACS. This positive effect of tACS on retention of acquired motor skill was accompanied by increased CMC and IMC in the beta band. Changes in CMC, but not IMC, were correlated with performance 1 and 7 days following practice.

### Retention of Improved Visuomotor Performance

The fundamental hypothesis, which has driven this study, is that retention of motor memory relies on neural activity in specific frequency bands in relation to acquisition of the task and that this activity-dependent consolidation may be enhanced by external stimulation at similar frequencies using tACS. It has been implied in a number of studies that the functional and structural neural changes that are responsible for learning and memory may be related to neural oscillations at different frequencies (Kandel et al. 2014; Andersen et al. 2017). Lasting improvements in visuomotor skill performance following motor practice (i.e., retention) requires structural changes in the neural circuitries involved in the execution of the skill (Luft and Buitrago 2005; Kantak and Winstein 2012). These structural changes are mediated by intracellular signaling factors that are transported to the nucleus where they activate transcription factors resulting in altered gene expression and protein synthesis (Asok et al. 2019), which allow prolonged effects on synaptic properties. Although this consolidation process is initiated immediately in relation to the training of the skill, the full process may continue for hours and possibly even days following the actual training period (Robertson et al. 2005). During this period, retention of the improved motor performance may be influenced by external factors that may either facilitate or impede the consolidation process (Brashers-Krug et al. 1996; Robertson et al. 2005). Learning a new task, low-frequency transcranial magnetic (Muellbacher et al. 2002; Lundbye-Jensen et al. 2011) or low frequency peripheral nerve stimulation (Lundbye-Jensen et al. 2011) in the consolidation period may disrupt retention of the improved performance, whereas sleep (Korman et al. 2007; King et al. 2017) or physical exercise (Roig et al. 2012; Thomas et al. 2016; Lundbye-Jensen et al. 2017) may facilitate retention. The findings in the present study suggest that tACS over the primary motor cortex at beta-band frequencies following visuomotor training may also facilitate consolidation and help to improve retention of the memory for the task at least 1 day following training. Krause et al. (Krause et al. 2016) similarly found that beta-frequency tACS may facilitate early consolidation of a finger sequence task.

Since we did not investigate the effect of other stimulation frequencies, we cannot determine from these experiments whether stimulation in the beta band was necessary in order to produce the observed effects on retention or superior to other types of stimulation. However, we chose stimulation in the beta band, since oscillations in that frequency band have been suggested from different lines of research to be involved in the consolidation of new learning (Khazipov et al. 2013; Minlebaev et al. 2011; Bönstrup et al. 2019). Following motor practice, primary motor cortical and spinal networks show common oscillations in the beta band reflected as an increase in beta-band coherence between cortex and muscle as well as between different motor unit populations within the trained muscle (Perez et al. 2006; Larsen et al. 2016). This increase in beta-band coherence may reflect an activity-dependent reorganization of neural connectivity in the corticospinal motor system related to the consolidation of improved motor performance. Beta-band activity also falls within the frequency range where animal experiments have documented that trains of stimuli may induce long-term potentiation (LTP) leading to manifest structural changes in neural connectivity (Nicoll 2017; Lømo 2018). While it is not possible to assess LTP in human experiments, LTP-like phenomena reflecting changes in network excitability, for example, for the primary motor cortex and the corticospinal pathway can be observed following motor skill practice (Perez et al. 2004) and these changes have been linked to consolidation and retention of skill in human experiments (Cantarero et al. 2013a, 2013b). It is therefore an exciting possibility that external stimulation at beta frequencies may influence the basic motor memory mechanisms that are responsible for the consolidation processes following practice. The observation by Krause et al. (2016) that transcranial direct current stimulation (tDCS) produced a similar effect on early consolidation following practice of a finger sequence task as tACS, however, suggests that a simple nonspecific increase of cortical excitability may also be involved. Indeed, increases of network excitability can also be seen following beta-band tACS (Wischnewski et al. 2019).

### Improved Motor Task Performance was Accompanied by Increased CMC and IMC

In contrast to previous studies, we did not observe any changes in beta-band CMC and IMC following visuomotor training (Perez et al. 2006; Larsen et al. 2016). We did however observe an increase in beta-band CMC and IMC in the tACS group at both retention tests. Furthermore, the overnight change in peak beta-band CMC showed a positive correlation to the total learning of the task tentatively suggesting that greater retention may be associated with increased beta-band oscillatory coupling in the corticospinal system leading to a more efficient consolidation processes following motor practice. This was not the case for the changes in alpha-band CMC, carefully suggesting some degree of frequency-specificity. It should be pointed out that there is considerable variability in the coherence measures that we obtained. This is evident from the measurements in the sham tACS control group that vary considerably at the different recording sessions. Part of the reason for this variability is that it is difficult to obtain EEG and EMG measurements from the exact same site at different sessions with several days in between. We took care to mark the position of the electrodes and ensured that the electrode impedance was similar in all cases, but we cannot fully exclude that the differences in coherence may have been influenced by differences in recording position or recording conditions. Furthermore, our correlational analyses tentatively tie together changes in CMC to motor performance, but these should be carefully interpreted because of the limited sample size. Potential associations between beta-band functional corticospinal coupling and changes in motor performance with motor practice should be further elucidated in future experiments.

### Limitations

It is a clear limitation of the study that we did not obtain recordings from more than a single electrode, since this prevents us from performing source localizing procedures. Availability of more than a bipolar electrode arrangement would also have made possible more sophisticated analyses for removal of sources of noise (eyeblinks and horizontal eye movements). It is also a limitation of the study that we only investigated the effect of tACS at a single frequency (peak CMC in the beta band) and at a single location. We therefore cannot conclude whether it was essential to stimulate at the peak frequency of (endogenous) corticomuscular rhythmicity or whether stimulation at other frequencies or electrode locations would have similar effects. Our electric field simulations, although based on a template brain, further hinted that other cortical regions in addition to the M1 leg area were affected by our tACS intervention. These regions include prefrontal and premotor areas, and the observed effects may thus also—at least in part—be mediated via stimulation induced effects in these areas. Conclusions relating to spatial specificity of the effects of tACS with the specified electrode montage and protocol should therefore be drawn cautiously.

The observed improvement in retention of motor performance following tACS was relatively small, although it reached statistical significance. This is not different from other studies where tACS and/or tDCS have been applied to facilitate motor performance, and there is a general concern in the field that the obtained effects are too small and variable to be of clinical significance for improvement of motor function in people with neurological disorders (Lefaucheur et al. 2017). This is probably the main reason why none of the available brain stimulation techniques have been applied routinely in the clinic to facilitate the effect of physiotherapy sessions or other neurorehabilitation interventions. In this context, it should, however, be pointed out that our study, and most other similar studies in the field, have only investigated the effect of a single session of tACS. It is not unlikely that repeated sessions involving a combination of motor practice followed by tACS over several weeks would produce much larger and clinically relevant effects. Studies that address this possibility should be strongly encouraged.

## Conclusion

This study shows that tACS applied at beta-band frequency improves visuomotor skill retention and facilitates CMC and IMC in the beta band. We propose that induced oscillatory activity in the corticospinal tract may be involved in facilitating consolidation of the skill and improving retention.

## Notes

**Tomofumi Yamaguchi**: Conceived and designed research, performed experiments, analyzed data, interpreted results of experiments, prepared figures, edited and revised the manuscript. Approved the final version of the manuscript.

**Christian Svane**: Performed experiments, analyzed data, interpreted results of experiments, prepared figures, edited and revised the manuscript. Approved the final version of the manuscript.

**Christian Riis Forman**: Performed experiments, analyzed data, interpreted results of experiments, prepared figures, edited and revised the manuscript. Approved the final version of the manuscript.

**Mikkel Malling Beck**: Conceived and designed research, analyzed data, interpreted results of experiments, prepared figures, drafted manuscript, edited and revised the manuscript. Approved the final version of the manuscript.

**Svend Sparre Geertsen**: Conceived and designed research, edited and revised the manuscript. Approved the final version of the manuscript.

**Jesper Lundbye-Jensen**: Conceived and designed research, interpreted results of experiments, edited and revised the manuscript. Approved the final version of the manuscript.

**Jens Bo Nielsen**: Conceived and designed research, supervised experiments, interpreted results of experiments, edited and revised the manuscript. Approved the final version of the manuscript.

The authors would like to acknowledge all the participants for their contribution to this study. *Conflicts of Interest:* None declared.

## Funding

Elsass Foundation (to Prof. Jens Bo Nielsen).

## Conflict of interest

None of the authors had any conflicts of interest. The study was made possible by a research grant to Jens Bo Nielsen from the Elsass foundation. The Elsass foundation is an independent, private foundation, which aims to support research related to Cerebral Palsy

## References

[ref1] Ali MM , Sellers KK, Frohlich F. 2013. Transcranial alternating current stimulation modulates large-scale cortical network activity by network resonance. J Neurosci. 33:11262–11275. 23825429 10.1523/JNEUROSCI.5867-12.2013 PMC6618612

[ref2] Amjad AM , Halliday DM, Rosenberg JR, Conway BA. 1997. An extended difference of coherence test for comparing and combining several independent coherence estimates: theory and application to the study of motor units and physiological tremor. J Neurosci Methods. 73:69–79. 9130680 10.1016/s0165-0270(96)02214-5

[ref3] Andersen N , Krauth N, Nabavi S. 2017. Hebbian plasticity in vivo: relevance and induction. Curr Opin Neurobiol. 45:188–192. 28683352 10.1016/j.conb.2017.06.001

[ref4] Asok A , Leroy F, Rayman JB, Kandel ER (2019) Molecular mechanisms of the memory trace. Trends Neurosci. 42:14–22. 30391015 10.1016/j.tins.2018.10.005 PMC6312491

[ref5] Bates D , Mächler M, Bolker BM, Walker SC. 2014. Fitting linear mixed-effects models using lme4. arXiv. 23:1–51.

[ref6] Belardinelli P , Laer L, Ortiz E, Braun C, Gharabaghi A. 2017. Plasticity of premotor cortico-muscular coherence in severely impaired stroke patients with hand paralysis. NeuroImage Clin. 14:726–733. 28409112 10.1016/j.nicl.2017.03.005 PMC5379882

[ref7] Bönstrup M , Iturrate I, Thompson R, Cruciani G, Censor N, Cohen LG. 2019. A rapid form of offline consolidation in skill learning. Curr Biol. 29:1346–1351.e4. 30930043 10.1016/j.cub.2019.02.049 PMC6482074

[ref8] Brashers-Krug T , Shadmehr R, Bizzi E. 1996. Consolidation in human motor memory. Nature. 382:252–255. 8717039 10.1038/382252a0

[ref9] Cantarero G , Lloyd A, Celnik P. 2013a. Reversal of long-term potentiation-like plasticity processes after motor learning disrupts skill retention. J Neurosci. 33:12862–12869. 23904621 10.1523/JNEUROSCI.1399-13.2013 PMC3728692

[ref10] Cantarero G , Tang B, O’Malley R, Salas R, Celnik P. 2013b. Motor learning interference is proportional to occlusion of LTP-like plasticity. J Neurosci. 33:4634–4641. 23486938 10.1523/JNEUROSCI.4706-12.2013 PMC3727291

[ref11] Chapman LJ , Chapman JP. 1987. The measurement of handedness. Brain Cogn. 6:175–183. 3593557 10.1016/0278-2626(87)90118-7

[ref12] Christiansen L , Madsen MJ, Bojsen-Møller E, Thomas R, Nielsen JB, Lundbye-Jensen J. 2018. Progressive practice promotes motor learning and repeated transient increases in corticospinal excitability across multiple days. Brain Stimul. 11:346–357. 29187320 10.1016/j.brs.2017.11.005

[ref13] Conway BA , Halliday DM, Farmer SF, Shahani U, Maas P, Weir AI, Rosenberg JR. 1995. Synchronization between motor cortex and spinal motoneuronal pool during the performance of a maintained motor task in man. J Physiol. 489:917–924. 8788955 10.1113/jphysiol.1995.sp021104 PMC1156860

[ref14] Espenhahn S , van Wijk BCM, Rossiter HE, de Berker AO, Redman ND, Rondina J, Diedrichsen J, Ward NS. 2019. Cortical beta oscillations are associated with motor performance following visuomotor learning. Neuroimage. 195:340–353. 30954709 10.1016/j.neuroimage.2019.03.079 PMC6547051

[ref15] Grosse P , Cassidy MJ, Brown P. 2002. EEG-EMG, MEG-EMG and EMG-EMG frequency analysis: physiological principles and clinical applications. Clin Neurophysiol. 113:1523–1531. 12350427 10.1016/s1388-2457(02)00223-7

[ref16] Halliday DM , Rosenberg JR, Amjad AM, Breeze P, Conway BA, Farmer SF. 1995. A framework for the analysis of mixed time series/point process data-theory and application to the study of physiological tremor, single motor unit discharges and electromyograms. Prog Biophys Mol Biol. 64:237–278. 8987386 10.1016/s0079-6107(96)00009-0

[ref17] Hansen NL , Nielsen JB. 2004. The effect of transcranial magnetic stimulation and peripheral nerve stimulation on corticomuscular coherence in humans. J Physiol. 561:295–306. 15358809 10.1113/jphysiol.2004.071910 PMC1665343

[ref18] Hothorn T , Bretz F, Westfall P. 2008. Simultaneous inference in general parametric models. Biometrical J. 50:346–363. 10.1002/bimj.200810425 18481363

[ref19] Kandel ER , Dudai Y, Mayford MR. 2014. The molecular and systems biology of memory. Cell. 157:163–186. 24679534 10.1016/j.cell.2014.03.001

[ref20] Kantak SS , Winstein CJ. 2012. Learning-performance distinction and memory processes for motor skills: a focused review and perspective. Behav Brain Res. 228:219–231. 22142953 10.1016/j.bbr.2011.11.028

[ref21] Khazipov R , Minlebaev M, Valeeva G. 2013. Early gamma oscillations. Neuroscience. 250:240–252. 23872391 10.1016/j.neuroscience.2013.07.019

[ref22] King BR , Hoedlmoser K, Hirschauer F, Dolfen N, Albouy G. 2017. Sleeping on the motor engram: the multifaceted nature of sleep-related motor memory consolidation. Neurosci Biobehav Rev. 80:1–22. 28465166 10.1016/j.neubiorev.2017.04.026

[ref23] Korman M , Doyon J, Doljansky J, Carrier J, Dagan Y, Karni A. 2007. Daytime sleep condenses the time course of motor memory consolidation. Nat Neurosci. 10:1206–1213. 17694051 10.1038/nn1959

[ref24] Krause V , Meier A, Dinkelbach L, Pollok B (2016) Beta band Transcranial alternating (tACS) and direct current stimulation (tDCS) applied after initial learning facilitate retrieval of a motor sequence. Front Behav Neurosci. 10:1–10. 26834593 10.3389/fnbeh.2016.00004 PMC4722123

[ref25] Larsen LH , Jensen T, Christensen MS, Lundbye-Jensen J, Langberg H, Nielsen JB. 2016. Changes in corticospinal drive to spinal motoneurones following tablet-based practice of manual dexterity. Physiol Rep. 4:e12684. 26811055 10.14814/phy2.12684 PMC4760389

[ref26] Lefaucheur JP , et al. (2017) Evidence-based guidelines on the therapeutic use of transcranial direct current stimulation (tDCS). Clin Neurophysiol. 128:56–92. 27866120 10.1016/j.clinph.2016.10.087

[ref27] Lømo T . 2018. Discovering long-term potentiation (LTP) – recollections and reflections on what came after. Acta Physiol. 222:1–22. 10.1111/apha.12921 28719040

[ref28] Luft AR , Buitrago MM (2005) Stages of motor skill learning. Mol Neurobiol. 32:205–216. 16385137 10.1385/MN:32:3:205

[ref29] Lundbye-Jensen J , Petersen TH, Rothwell JC, Nielsen JB. 2011. Interference in ballistic motor learning: specificity and role of sensory error signals. PLoS One. 6:e17451. 21408054 10.1371/journal.pone.0017451 PMC3052297

[ref30] Lundbye-Jensen J , Skriver K, Nielsen JB, Roig M, Hospital JR. 2017. Acute exercise improves motor memory consolidation in preadolescent children. Front Hum Neurosci. 11:1–10. 28473761 10.3389/fnhum.2017.00182 PMC5397400

[ref31] Masakado Y , Nielsen JB. 2008. Task-and phase-related changes in cortico-muscular coherence. Keio J Med. 57:50–56. 18382125 10.2302/kjm.57.50

[ref32] Minlebaev M , Colonnese M, Tsintsadze T, Sirota A, Khazipov R. 2011. Early gamma oscillations synchronize developing thalamus and cortex. Science. 334:226–229. 21998388 10.1126/science.1210574

[ref33] Muellbacher W , Ziemann U, Wissel J, Dang N, Kofler M, Facchini S, Boroojerdi B, Poewe W, Hallett M. 2002. Early consolidation in human primary motor cortex. Nature. 415, 640–4. 10.1038/nature712 11807497

[ref34] Nicoll RA (2017) Review a brief history of long-term potentiation. Neuron. 93:281–290. 28103477 10.1016/j.neuron.2016.12.015

[ref35] Omlor W , Patino L, Hepp-Reymond MC, Kristeva R. 2007. Gamma-range corticomuscular coherence during dynamic force output. Neuroimage. 34:1191–1198. 17182258 10.1016/j.neuroimage.2006.10.018

[ref36] Perez MA , Lundbye-Jensen J, Nielsen JB. 2006. Changes in corticospinal drive to spinal motoneurones following visuo-motor skill learning in humans. J Physiol. 573:843–855. 16581867 10.1113/jphysiol.2006.105361 PMC1779755

[ref37] Perez MA , Lungholt BKS, Nyborg K, Nielsen JB. 2004. Motor skill training induces changes in the excitability of the leg cortical area in healthy humans. Exp Brain Res. 159:197–205. 15549279 10.1007/s00221-004-1947-5

[ref38] Petersen TH , Willerslev-Olsen M, B a C, Nielsen JB. 2012. The motor cortex drives the muscles during walking in human subjects. J Physiol. 590:2443–2452. 22393252 10.1113/jphysiol.2012.227397 PMC3424763

[ref39] Robertson EM , Press DZ, Pascual-Leone A. 2005. Off-line learning and the primary motor cortex. J Neurosci. 25:6372–6378. 16000627 10.1523/JNEUROSCI.1851-05.2005 PMC6725279

[ref40] Roig M , Skriver K, Lundbye-Jensen J, Kiens B, Nielsen JB. 2012. A single bout of exercise improves motor memory. PLoS One. 7:e44594. 22973462 10.1371/journal.pone.0044594 PMC3433433

[ref41] Spedden ME , Jensen P, Terkildsen CU, Jensen NJ, Halliday DM, Lundbye-Jensen J, Nielsen JB, Geertsen SS. 2019. The development of functional and directed corticomuscular connectivity during tonic ankle muscle contraction across childhood and adolescence. Neuroimage. 191:350–360. 30818025 10.1016/j.neuroimage.2019.02.054

[ref42] Spedden ME , Nielsen JB, Geertsen SS (2018) Oscillatory corticospinal activity during static contraction of ankle muscles is reduced in healthy old versus young adults. Neural Plast. 2018:1–13. 10.1155/2018/3432649 PMC5944232 29853842

[ref43] Thomas R , Beck MM, Lind RR, Johnsen LK, Geertsen SS, Christiansen L, Ritz C, Roig M, Lundbye-Jensen J. 2016. Acute exercise and motor memory consolidation: the role of exercise timing. Neural Plast. 1–25 https://www.hindawi.com/journals/np/2016/6205452/. 10.1155/2016/6205452 PMC4947505 27446616

[ref44] Turi Z , Ambrus GG, Janacsek K, Emmert K, Hahn L, Paulus W, Antal A. 2013. Both the cutaneous sensation and phosphene perception are modulated in a frequency-specific manner during transcranial alternating current stimulation. Restor Neurol Neurosci. 31:275–285. 23478342 10.3233/RNN-120297

[ref45] Wischnewski M , Schutter DJLG, Nitsche MA (2019) Effects of beta-tACS on corticospinal excitability: a meta-analysis. Brain Stimul. 12:1381–1389. 31405789 10.1016/j.brs.2019.07.023

